# Shotgun Sequencing Revealed the Microbiota of Zea mays Rhizosphere of a Former Grassland and an Intensively Cultivated Agricultural Land

**DOI:** 10.1128/MRA.01058-20

**Published:** 2020-12-03

**Authors:** Olubukola Oluranti Babalola, Chinenyenwa Fortune Chukwuneme, Ayansina Segun Ayangbenro

**Affiliations:** a Food Security and Safety Niche, Faculty of Natural and Agricultural Sciences, North-West University, Mmabatho, South Africa; Indiana University, Bloomington

## Abstract

Land use is a major factor contributing to the differences in soil microbial assemblages. Despite the importance of microbial communities on crop health and productivity, a knowledge gap exists on the effects of land use change on microbial functions in the rhizosphere. This data set presents the metagenomic data from two maize fields in South Africa with different agricultural histories. It provides an opportunity for modeling microbes with beneficial functions that could enhance crop productivity.

## Abstract (precis)

Land use is a major factor contributing to the differences in soil microbial assemblages. Despite the importance of microbial communities on crop health and productivity, a knowledge gap exists on the effects of land use change on microbial functions in the rhizosphere.

## ANNOUNCEMENT

To increase food production, land has continuously been converted to agricultural land (1), prompting research on land use conversion implications on the soil microbiota aiding plant growth. Here, we present the data from a former grassland in Ventersdorp, South Africa, and land in Mafikeng, South Africa, with over 30 years of intensive cropping.

Soils tightly adhered to the plants’ roots were collected from Ventersdorp (F1), at 26°19′38″S and 26°53′18″E, and Mafikeng (F2), at 25°48′00″S and 25°38′21″E. Samples were collected from four points in each field (F1, GZ1 to GZ4; F2, AG1 to AG2). Soil samples were sieved and homogenized, and whole microbial DNA was extracted from 5 g of each sample using a DNeasy PowerMax soil kit (Qiagen, Denmark) following the manufacturer’s instructions. This study’s data sets are whole-metagenome shotgun-sequencing products at the Molecular Research Laboratory (MR DNA, Shallowater, TX, USA). Metagenomic DNA libraries were set using the Nextera DNA Flex library preparation kit (Illumina) according to the manufacturer’s guidelines. The initial DNA concentration was determined using the Qubit double-stranded DNA (dsDNA) high-sensitivity (HS) assay kit (Life Technologies), and samples were cleaned with a DNeasy PowerClean Pro cleanup kit (Qiagen). The samples were further subjected to simultaneous fragmentation, and adapter sequences were added and used in a limited-cycle PCR. After that, unique indices were added, and final library concentrations were determined using a Qubit dsDNA HS assay kit (Life Technologies), while the average size of the library was measured using an Agilent 2100 bioanalyzer. The libraries were pooled and diluted to 0.6 nM and paired-end sequenced for 300 cycles using the NovaSeq system (Illumina). Analysis and annotation of output data were performed in the metagenomics rapid annotation (MG-RAST) online server (2, 3) using default parameters. Following quality control (QC), sequences were annotated using the BLAT algorithm (4, 5) against the M5nr database (6), which offers nonredundant integration of numerous databases.

Taxonomic classification at the domain level revealed that in the GZ samples, 98.73%, 0.76%, 0.41%, and 0.02% of sequences were assigned to bacteria, eukaryotes, archaea, and viruses, respectively, while in the AG samples, 98.09%, 1.51%, 0.35%, and 0.01% of sequences were assigned to bacteria, eukaryotes, archaea, and viruses, respectively. We also observed that the bacterial phyla *Actinobacteria* and *Proteobacteria* dominated the samples.

The functional categories using SEED subsystems showed that the sequences were linked with various metabolisms, including carbohydrates, amino acids and derivatives, stress response, etc. This shotgun metagenomic study revealed important metabolic and functional potentials of microbial inhabitants of the environments. Table 1 presents the general statistics and sequence quality information of the samples.

**TABLE 1:** General statistics and quality of sequences from the MG-RAST database

Sample	Avg no. of raw sequence reads	Avg no. of reads after quality control	Avg no. of rRNA genes present	Avg no. of predicted proteins with known functions	Avg no. of predicted proteins with unknown functions
GZ	8,224,132	7,168,268	7,668	2,458,152	3,822,426
AG	5,514,523	4,849,854	7,775	1,812,925	2,367,011

### Data availability.

We deposited the data files (reads in FASTQ format) at the NCBI SRA database under BioProject accession number PRJNA649682. The annotated data after quality control can be found in the MG-RAST database with the accession numbers mgm4898320.3, mgm4898326.3, mgm4898324.3, and mgm4898327.3 for F1 samples and mgm4898321.3, mgm4898316.3, mgm4898317.3, and mgm4898323.3 for F2 samples.
